# One-step, efficient and sustainable microwave-assisted biodiesel production using a sulfonated porous organic polymer catalyst

**DOI:** 10.1039/d5ra07846f

**Published:** 2025-11-27

**Authors:** Biman Kaushik, Shikhasmita Das, Sanjay Basumatary, Ruma Rano, Hui Li, Jasha Momo H. Anal, Gopinath Halder, Samuel Lalthazuala Rokhum

**Affiliations:** a Department of Chemistry, National Institute of Technology Silchar Silchar 788010 Assam India rokhum@che.nits.ac.in; b Department of Chemistry, Bodoland University Kokrajhar 783370 Assam India; c School of Thermal Engineering, Shandong Jianzhu University Jinan 250101 PR China; d Natural Products and Medicinal Chemistry Division, CSIR-Indian Institute of Integrative Medicine Jammu 180001 Jammu and Kashmir India; e Department of Chemical Engineering, National Institute of Technology Durgapur Durgapur 713209 West Bengal India

## Abstract

The transition toward renewable fuels requires robust, recyclable, and eco-friendly catalysts for biodiesel synthesis. Here, we reported the synthesis process of a sulfonated covalent triazine framework-based porous organic polymer (CTF-POP-SO_3_H) and the microwave-assisted esterification of oleic acid with methanol using a heterogeneous CTF-POP-SO_3_H catalyst. The catalyst exhibited a high biodiesel conversion of 96.61% under optimized conditions (methanol-to-oil ratio, 20 : 1; catalyst loading, 8 wt%; reaction time, 50 min; temperature, 100 °C) with product formation confirmed by ^1^H NMR, ^13^C NMR, and GC analyses. Comprehensive characterization of the catalyst was conducted using FTIR, BET, TGA, XRD, XPS, and SEM-EDX-MAPPING. The presence of acidic sites (–SO_3_H) is confirmed by acid–base titration, which is well aligned with SEM-EDX-MAPPING. Kinetic evaluation revealed a low activation energy of 24.52 kJ mol^−1^, while thermodynamic analysis indicated an endothermic process. Importantly, the catalyst retained over 80% of its activity after five successive cycles, confirming its durability and reusability. These results highlight that the sulfonated porous organic polymer is an efficient and sustainable catalyst for biodiesel production, providing an eco-friendly pathway aligned with global clean energy targets.

## Abstract 2

This study reports that a sulfonated covalent triazine framework porous organic polymer catalyst enables high biodiesel conversion through efficient microwave-assisted esterification.

## Graphical abstract

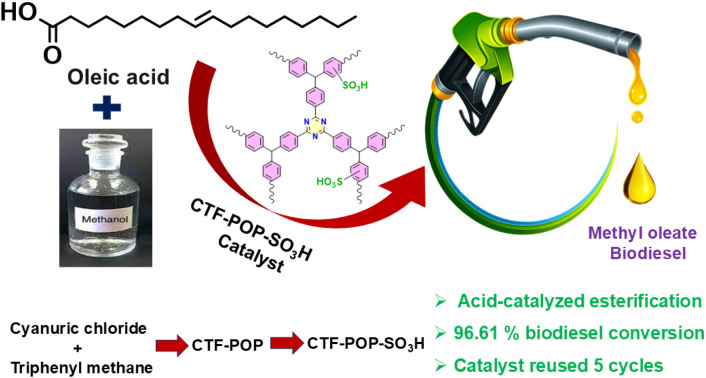

## 1. Introduction

The United States is currently the world's leading energy consumer, followed by China and India, with fossil fuels still accounting for about 88% of India's overall energy consumption.^1^ The decreasing supply of fossil fuel resources, rising global energy demand, and rising environmental concerns have intensified the search for renewable, sustainable, and environmentally friendly energy alternatives. Among these, biodiesel has emerged as a promising candidate produced from triglycerides and free fatty acids available in renewable substrates such as plant-based oils, animal fats and residual cooking oil.^2,3^ Its multifaceted benefits, such as biodegradability, non-toxicity, environmental compatibility, and the ability to lower greenhouse gas emissions make biodiesel a compelling alternative to conventional fossil fuels.^4^ In addition, biodiesel exhibits superior fuel properties, including better lubrication, a greater cetane number, and favourable combustion properties similar to petroleum diesel.^5,6^ Usually made by esterifying FFAs or *trans*-esterifying triglycerides, biodiesel is renowned for burning cleaner than regular diesel. This procedure uses either acid or base catalysts and short-chain alcohols, most frequently methanol.^7^ More than 95% of biodiesel is produced from edible oils, a practice that can exacerbate food shortages and drive-up food prices, particularly in developing countries.^8^ In this work, we took oleic acid as a model oil substrate owing to its abundance in second-generation biodiesel feedstocks such as waste cooking oil, jatropha oil, and Karanja oil.^2,9^

Apart from feedstocks, the choice of catalyst plays a pivotal role in biodiesel production, as it directly influences both process efficiency and overall economic feasibility.^10,11^ Common practices for biodiesel synthesis predominantly utilize homogeneous acid or base catalysts due to simplicity and high activity. While effective, these catalysts suffer from significant limitations, including complex separation processes and environmental concerns.^12^ As a result, there has been a growing focus on heterogeneous solid acid catalysts, which provide benefits like improved recyclability, simplified recovery and customizable surface characteristics.^2^ Since base-catalyzed transesterification often leads to soap formation and complicates wastewater treatment, esterification using an acid catalyst typically with methanol or glycerol is preferred when the feedstock contains a high concentration of FFAs.^13,14^ In the presence of homogeneous catalysts like H_2_SO_4_ and H_3_PO_4,_ ester production *via* esterification or transesterification requires high temperature, expensive equipment, and has limitations in the reusability of the catalyst.^15–17^ Because of their corrosive nature, these minerals must be neutralized following the reaction. Metal alkoxides such as NaOH or KOH, which are used for esterification and transesterification, are also not suitable as they are prone to saponification and require a lot of water to wash byproducts.^18,19^ These problems can be solved by the recently introduced solid heterogeneous catalysts, porous organic polymers (POPs).

One extremely versatile class of lightweight materials with catalytic activity is represented by POPs. They are made entirely of organic materials joined by strong covalent bonds.^20^ These materials are characterized by their permanent porosity, tunable pore size, and chemical functionality, which can be tailored by judicious choice of monomers and synthetic strategies.^21^ With applications ranging from gas storage to separation procedures, heterogeneous catalysis, energy retention, sensing technologies, and a variety of optoelectronic applications, POPs have drawn a lot of attention due to their versatility.^22^ Sudipta and co-workers^23^ developed a sulfonated, hyper-cross-linked pyrene-based porous organic polymer derived from carbazole, featuring a super microporous architecture. This metal-free heterogeneous catalyst demonstrated effective biodiesel production at room temperature over 10 h.^23^ Although it is room-temperature catalysis, such long durations limit industrial feasibility. Another hyper-cross-linked KNO_3_ impregnated porous polymer reported by Señorans and co-workers delivered a 99.9% biodiesel yield initially but suffered a rapid decline to 42% after the first reuse owing to potassium leaching.^24^ A PPM-SO_3_H porous polymer monolith catalyst was studied for biodiesel synthesis, but the reaction required nearly 8 h to reach high conversion, reflecting relatively slow kinetics under conventional conditions.^25^ The CTF-POP-SO_3_H catalyst, on the other hand, works much better. It can produce high yield of biodiesel in just 50 min of microwave irradiation, has very little active-site leaching, and maintains stability for multiple reuse cycles, showing faster kinetics and better stability than other porous polymer catalysts that have been reported. Compared to conventional thermal methods, microwave-assisted transesterification or esterification, a non-contact heating method, significantly enhances biodiesel synthesis, yielding higher product quality and significantly faster reaction rates.^26^ Microwave assisted reaction was done by using methanol and oleic acid oil to produce methyl oleate. Efficiency of chemical transformations largely depends on how effectively heat is delivered to the reactants. Traditional heating methods often involve prolonged reaction times to reach optimal conversion of oil into biodiesel.^27^

Although significant progress has been achieved in microwave-assisted biodiesel synthesis, most reported catalysts are still limited to sulfonated carbons, zeolites, and metal oxides, while only a few studies have explored –SO_3_H functionalized covalent triazine framework-based POPs specifically designed for microwave driven biodiesel production. To the best of our knowledge, the use of CTF-POP-SO_3_H as a solid acid catalyst for biodiesel synthesis represents a novel approach. In this work, we report the first synthesis of CTF-POP *via* a Friedel–Crafts reaction, followed by chlorosulfonic acid sulfonation at room temperature, yielding an efficient heterogeneous catalyst that promotes the microwave-assisted esterification of oleic acid with a faster reaction rate compared with previously reported systems.

## 2. Materials and methods

### 2.1. Chemicals used

Cyanuric chloride, triphenylmethane, and dichloromethane (DCM) were purchased from Sisco research laboratories. Analytical-grade methanol, ethyl acetate, and chlorosulphonic acid were bought from Merck, and ibuychemikals delivered anhydrous aluminium chloride. All reagents were used as supplied, without further purification.

### 2.2. Catalyst preparation

#### 2.2.1. Synthesis of CTF-POP

**Fig. 1:**
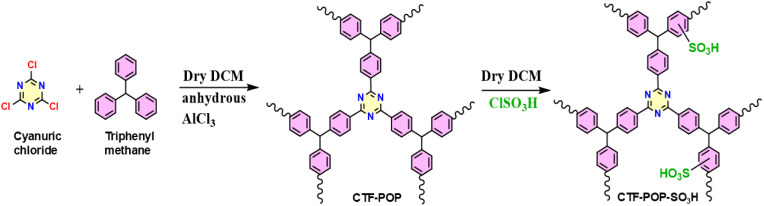
Synthetic procedure of CTF-POP-SO_3_H catalyst.

Anhydrous AlCl_3_ (19.5 mmol, 2.6 g) was dissolved in dry DCM (25 mL, 99%) in inert condition, and then cyanuric chloride (1 g, 5.4 mmol) was mixed slowly and stirred for 1 h at 25 °C as shown in the first step of Fig. 1.^28^ Next, a triphenylmethane (1.3 g, 5.3 mmol) solution was prepared in dry DCM (25 mL, 99%) and added dropwise to the solution.^28^ The reaction mixture was stirred at 38 °C for 16 h, after which the yellowish solid was extracted from the solution and washed three times with water, DCM, THF, and Methanol. The formed solid (CTF-POP) was placed in an oven for drying at 70 °C for 12 h.

#### 2.2.2. Synthesis of CTF-POP-SO_3_H

0.5 g of CTF-POP was dispersed in dry DCM, and 1.5 mL of chlorosulfonic acid (ClSO_3_H) was added dropwise using a dropping funnel to the suspension under a cooled environment (ice bath <5 °C) to control exothermic reaction as shown in the second step of Fig. 1. The mixture was then stirred for 24 h at room temperature (25 °C). The synthesized solid CTF-POP-SO_3_H was subsequently washed with ice cold water and methanol and subjected to vacuum drying at 90 °C for 4 h.

### 2.3. Characterization of catalyst

IR spectra were recorded in the range of 400–4000 cm^−1^ using a Bruker 3000 Hyperion FTIR spectrometer (Bruker, Germany). Quantachrome (Anton Paar) Surface Area & Pore Size Analyzer was used to determine the surface area and total pore volume (BET analysis). TGA was carried out using a Mettler Toledo TGA/DSC 1 STARe System, which has an upper temperature limit of 1000 °C. Under a constant nitrogen flow, the analysis was conducted over a temperature range of 20–700 °C at a heating rate of 5 °C min^−1^. Catalyst's morphology was examined using a FEI Quanta FEG 200F scanning electron microscope, equipped with a field emission gun (FEG) assembly featuring a Schottky emitter (operating from −200 V to 30 kV) and capable of delivering beam currents greater than 100 nA. EDX and elemental mapping features for comprehensive characterization were done. An X'Pert Pro diffractometer with Cu Kα radiation was used to obtain XRD patterns. A K-Alpha XPS spectrometer (Thermo Scientific NEXSA Surface Analysis) equipped with a monochromatic Al Kα X-ray source was used to perform XPS.

### 2.4. Biodiesel production *via* esterification of oleic acid using CTF-POP-SO_3_H catalyst

Methanol (0.648 g, 20 mmol), oleic acid (0.281 g, 1 mmol), and CTF-POP-SO_3_H (22 mg, 8 wt% relative to oleic oil) were mixed in a microwave reaction tube of 10 mL having a small magnetic bead. A Discover SP Microwave System was used to microwave the reaction mixture for 50 min at 100 psi, 100 °C and 50 W. After completion, the mixture was allowed to cool to room temperature, and the catalyst was removed by centrifugation. The resulting filtrate was concentrated under reduced pressure employing a rotary evaporator to extract out excess methanol, affording the crude methyl oleate (biodiesel) product. The formed ester was confirmed by thin-layer chromatography.

### 2.5. Methyl oleate (biodiesel) characterization

Using a Bruker Avance 500 MHz spectrometer, ^13^C NMR and ^1^H NMR spectroscopy was utilized to characterize the esterified compound. Gas chromatography combined with high-resolution mass spectrometry was performed using an Agilent 7890 system. The injector and detector temperatures were kept at 200 °C and 300 °C, respectively, while the GC oven temperature was set to range from 60 °C to 280 °C. We calculated biodiesel conversion from fatty acid using eqn (1) and (3)^29^ and yield by using eqn (2),^30^ respectively.1
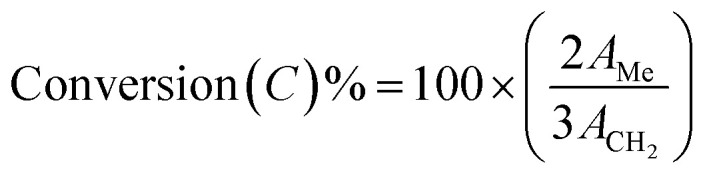
where *A*_Me_ is the integral area portion of –OCH_3_ and *A*_CH_2__ the area of –CH_2_2
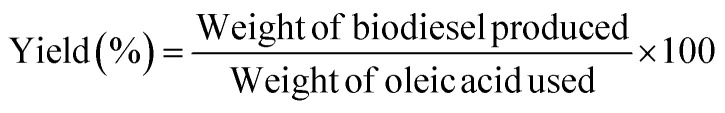
3
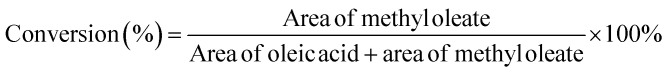


### 2.6. Acid density calculation

The density of the sulfonic acid group was determined *via* acid–base titration following the method reported by Ning and Niu,^31^ with slight modifications. In a typical procedure, 35 mg of the catalyst sample was dispersed in 20 mL of a supersaturated NaCl solution and stirred for 24 hours to allow for complete ion exchange. The resulting suspension was then filtered using quantitative filter paper to separate the solid. The filtrate was titrated with a standardized 0.02 M NaOH solution using phenolphthalein as an indicator. The sulfonic acid group density (*C*_SO_3_H_ in mmol g^−1^) was calculated using eqn (4).4
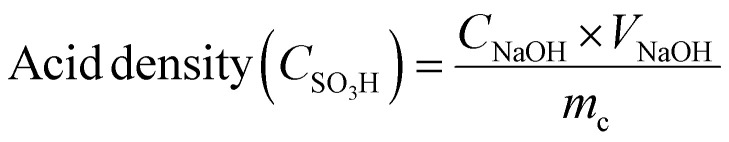
where *m*_c_ denotes mass (in gm) of catalyst taken; *C*_SO_3_H_ denotes acid density of sulphonic group in mmol g^−1^; *V*_NaOH_ represents amount of NaOH required (in mL); *C*_NaOH_ stands for concentration of NaOH (mol L^−1^).

### 2.7. Reaction kinetics

Oleic acid esterification reaction proceeds under pseudo-first-order conditions due to the significant excess of methanol, which permits the neglect of the reverse reaction. As a result, the reaction rate can be written as follows:5
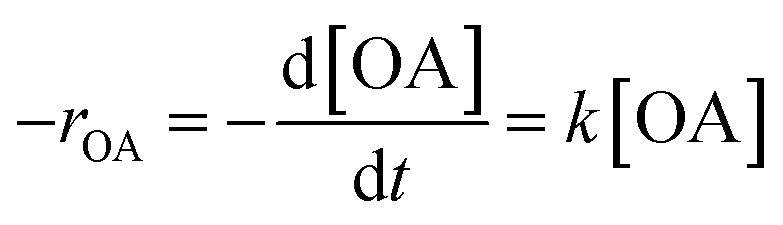
where [OA] is the oleic acid concentration and *k* is the pseudo-first-order rate constant. The integrated first-order rate law can be used to determine *k* by tracking the transformation of oleic acid (or the formation of methyl oleate) at different time intervals in eqn (6).

6 ln(1 − *X*) = −*kt*

where *X* represents the oleic acid fractional conversion at time *t*. The Arrhenius equation eqn (7) was used to calculate the activation energy (*E*_a_) based on the temperature dependence of *k* over the range of 40–100 °C.7
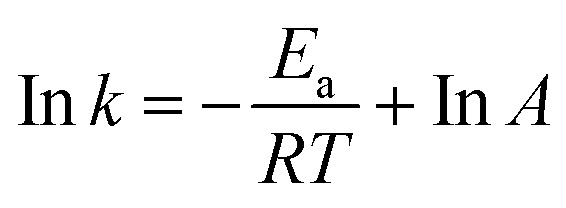
Here, *R* is the universal gas constant (8.314 × 10^−3^ kJ mol^−1^ K^−1^), *T* is the absolute temperature (in K), and *A* is frequency factor and *E*_a_ is the activation energy of the reaction. Furthermore, the esterification of oleic acid using the CTF-POP-SO_3_H catalyst was examined at different temperatures, leading to the assessment of thermodynamic features such as enthalpy change (Δ*H*^‡^), entropy change (Δ*S*^‡^) and Gibbs free energy (Δ*G*^‡^) by using Eyring–Polanyi eqn (8) and Gibbs free energy eqn (9).8
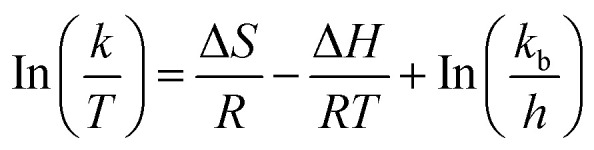

9 Δ*G*^‡^ = Δ*H*^‡^ and Δ*S*^‡^

In this context, *h* represents Planck's constant (6.626 × 10^−34^ J s), and *k*_b_ is the Boltzmann constant (1.38 × 10^−23^ J K^−1^).

## 3. Results and discussions

### 3.1. Catalyst characterisation

**Fig. 2:**
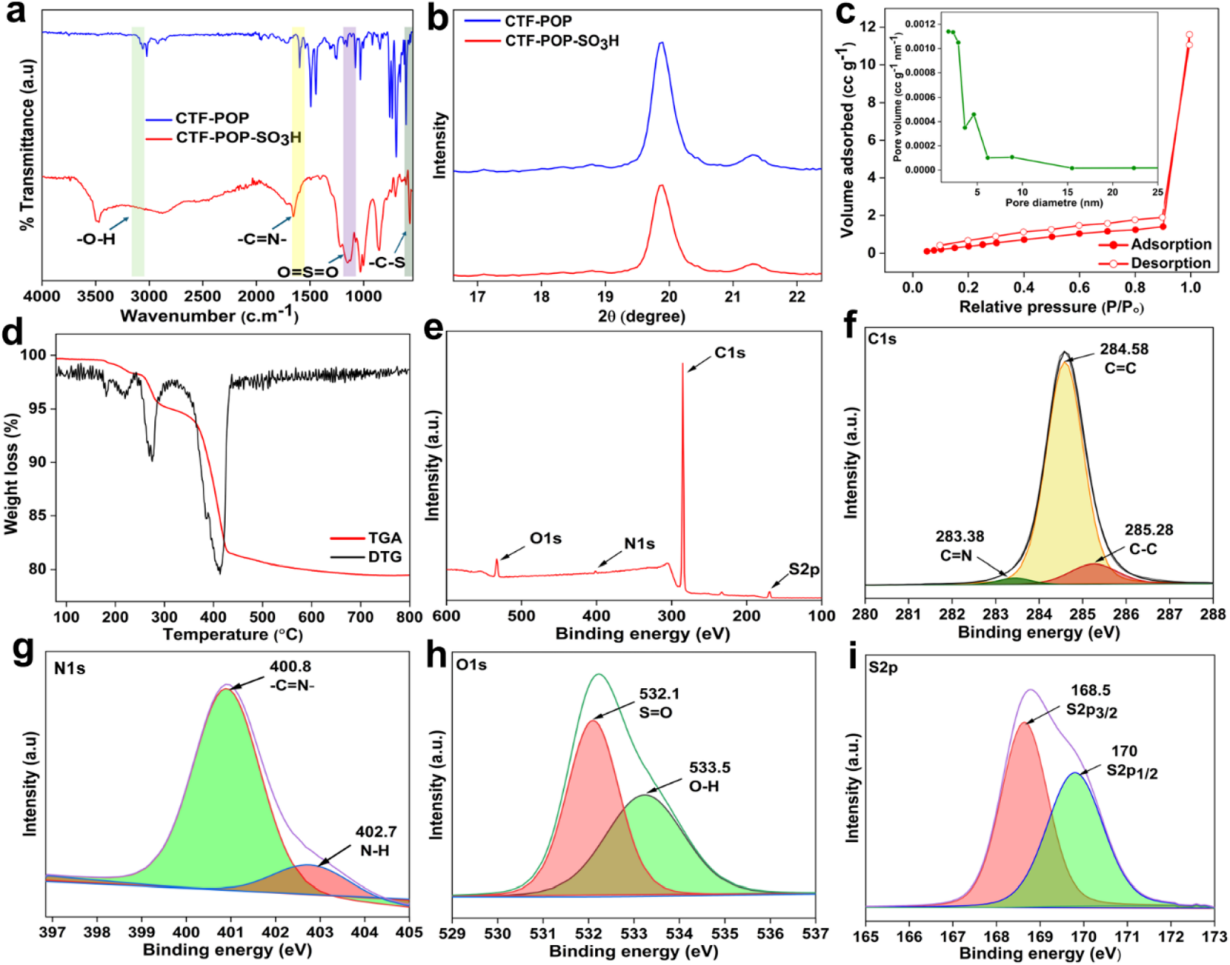
(a) FTIR and (b) XRD of CTF-POP (blue) and CTF-POP-SO_3_H (red); (c) BET (pore size distribution) and (d) TGA-DTG of CTF-POP; (e) XPS survey spectrum of CTF-POP-SO_3_H; for the CTF-POP-SO_3_H catalyst, deconvoluted XPS signals (red, green, yellow, orange) data for the (f) C 1s, (g) N 1s, (h) O 1s, and (i) S 2p regions.

Following synthesis and post-synthetic sulfonation, CTF-POP-SO_3_H was characterized using various analytical techniques and compared to its non-functionalized CTF-POP. The successful introduction of the sulfonic acid group was confirmed by FTIR spectroscopy in Fig. 2a. Characteristic O=S=O stretching vibrations were observed at 1149 and 1028 cm^−1^, while C–S stretching appeared at 568 cm^−1^, indicating the covalent incorporation of sulfonic acid moieties into the polymer backbone. Additionally, a wide absorption band spanning the range of 3500–2750 cm^−1^, centered around 3000 cm^−1^ attributed to O–H stretching vibrations of the sulfonic acid group, further confirming the presence of –SO_3_H functionalities.^2,28,32^

The XRD of CTF-POP-SO_3_H in Fig. 2b verified the existence of a distinctive peak position that was in good alignment with the CTF-POP appeared at 2*θ* = 20 °C, consistent with previous studies.^28^ However, it was found that the catalyst's intensity had somewhat decreased, which might have been caused by the material's pore blockage.

The catalyst specific surface area and pore volume were determined by N_2_ adsorption/desorption isotherm, which is given in Fig. 2c. The surface area of CTF-POP-SO_3_H in Fig. 2c and recovered catalyst R-CTF-POP-SO_3_H given in Fig. S1 (refer to SM) were found to be 2.74 m^2^ g^−1^ and 2 m^2^ g^−1^ respectively while their pore volumes were 0.019 cm^3^ g^−1^ and 0.006 cm^3^ g^−1^, respectively. The analysis of the pore size distribution showed pore diameters of 1.806 nm and 2.325 nm for CTF-POP-SO_3_H and R-CTF-POP-SO_3_H respectively. The slight decrease in surface area observed in the catalyst recovered after the fifth cycle is attributed to partial pore filling by methanol. When catalyst activity is high and BET is low, activity takes precedence over surface area.^33^

TGA analysis under a nitrogen atmosphere was employed to evaluate the thermal robustness of the prepared catalyst. A distinct weight loss profile was observed for CTF-POP-SO_3_H in the temperature range of 251–450 °C in Fig. 2d. The TGA-DTG curve of CTF-POP-SO_3_H showed an initial weight loss of approximately 4% between 100–220 °C, which can be resulted from the loss of moisture from the polymer framework. A subsequent weight loss of 7% occurring between 220–360 °C which can be associated with the breakdown of –SO_3_H group. Further weight loss can be attributed to the framework breakdown between 360–450 °C. The high thermal stability observed beyond this range can be due to the presence of strong aromatic C–C bonds in the polymer backbone.^34,35^

XPS provided detailed insights into the surface composition and binding energies of CTF-POP-SO_3_H. The wide-scan XPS survey showed distinct peaks corresponding to C 1s, N 1s, O 1s, and S 2p in CTF-POP-SO_3_H given in Fig. 2e. In the C 1s spectrum of Fig. 2f, a sharp peak at 284.58 eV corresponds to C=C bonds, while the peaks at 283.38 eV and 285.28 eV are attributed to aromatic triazine (–C=N–) and C–C bonds, respectively. Nitrogen's presence is verified by the N 1s signal in Fig. 2g at 400.8 eV consistent with the triazine structure of the CTF backbone, and the 402.7 peak is for the interaction of the –SO_3_H group.^2^ The O 1s spectrum in Fig. 2h exhibited two signals at 532.1 and 533.5 eV resulting from S=O and O–H bonds, respectively.^2^ The deconvoluted S 2p spectrum of CTF-POP-SO_3_H confirmed the existence of sulfur species in higher oxidation states, which results from the sulfonation of CTF-POP given in Fig. 2i. The binding energies at 170 and 168.5 eV were assigned to S 2p_1/2_ and S 2p_3/2_ respectively, indicating that sulfur exists exclusively as –SO_3_H groups.^34–36^ The CTF-POP framework's successful sulfonation is amply demonstrated by the XPS results.

**Fig. 3:**
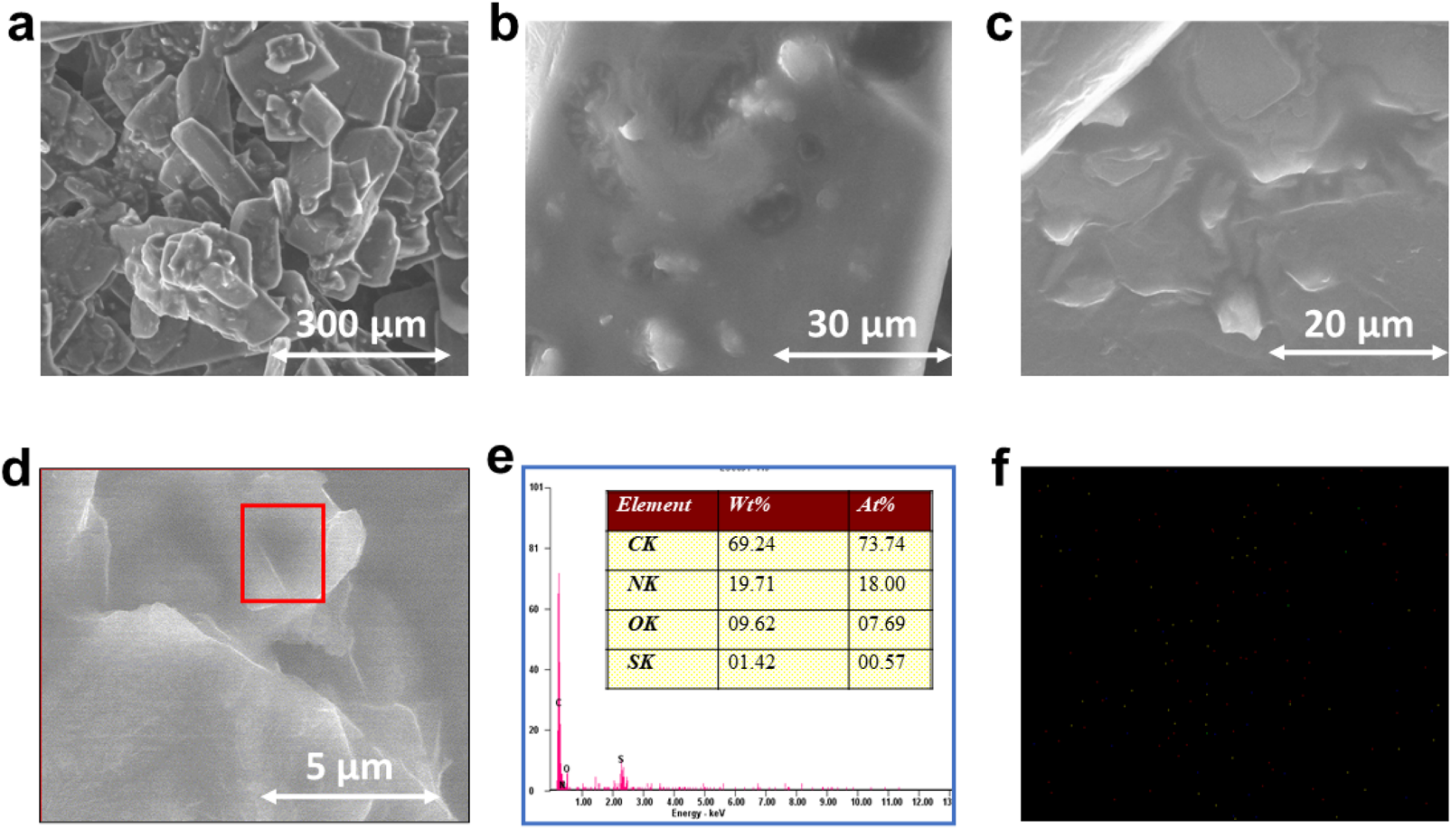
SEM images of (a–d) CTF-POP-SO_3_H along with the EDS data (e) for the region highlighted in the red box in (d) and (f) overall elemental mapping of CTF-POP-SO_3_H.

The morphology of CTF-POP-SO_3_H was examined using SEM as shown in Fig. 3. CTF-POP-SO_3_H exhibited a relatively flat surface. In contrast to the uniform morphology of the recovered catalyst, which is displayed in Fig. S4 (refer to SM) as a result of the reduction of sulfur content in the five cycles of recovered catalyst, the SEM images revealed closely packed primary particles with minor surface irregularities brought on by the sulfonation process. EDX also presented in Fig. 3e, confirmed the presence of nitrogen (N), sulphur (S), oxygen (O), and carbon (C) with relative atomic wt. percentage of 18%, 0.57%, 7.69%, and 73.74%, respectively. Overall elemental mapping in Fig. 3f indicated distribution of S (yellow), O (blue), N (green), and C (red) throughout the CTF-POP-SO_3_H framework. The atomic weight percentage of sulphur in the recovered catalyst was examined by EDX analysis given in Fig. S4 (refer to SM) and shows a decrease in the sulphur content (0.30%). A sulfonic acid group density of 0.21 mmol g^−1^ was determined through acid–base titration using eqn (4) and can be related to the SEM-EDX weight percentage of the sulfonic group.

### 3.2. Characterization of methyl oleate biodiesel

**Fig. 4:**
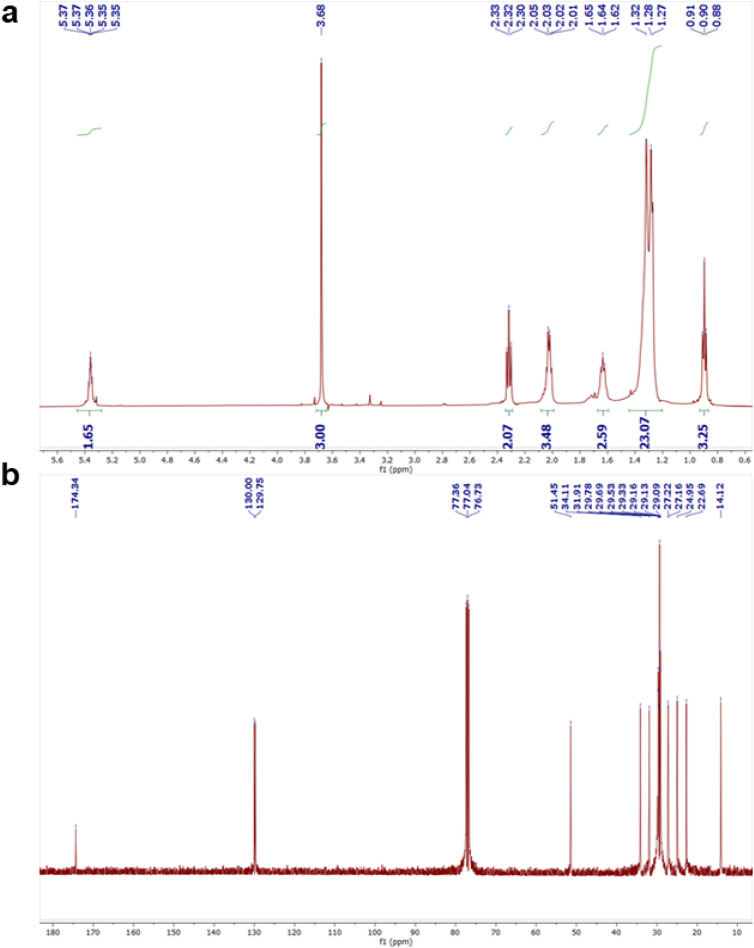
(a) ^1^H NMR (500 MHz, CDCl_3_, 27 °C) (b) ^13^C NMR (126 MHz, CDCl_3_, 27 °C) for methyl oleate produced using CTF-POP-SO_3_H catalyst.

^1^H-NMR spectroscopy was employed to determine the percentage conversion of biodiesel obtained from oleic acid using the CTF-POP-SO_3_H catalyst. As per previous studies Oleic acid and methyl oleate ^1^H spectra are basically identical, the key distinguishing signal being the new –OCH_3_ singlet at ∼3.68 ppm in methyl oleate. Fig. 4a presents the representative ^1^H-NMR spectrum (500 MHz, CDCl_3_, 27 °C) of biodiesel produced with the catalyst under optimized conditions (methanol-to-oil ratio, 20 : 1; catalyst loading, 8 wt%; reaction time, 50 min; temperature, 100 °C). The corresponding ^13^C-NMR spectrum (126 MHz, CDCl_3_, 27 °C) is shown in Fig. 4b. The transformation was quantitatively assessed using ^1^H NMR, where a singlet at 3.68 ppm attributed to the –OCH_3_ protons emerged following esterification. The existence of olefinic hydrogen was indicated by the presence of a multiplet at 5.36 ppm. Based on the integration of these NMR peaks, the transformation of oleic acid to methyl oleate was determined to be 96.61% using eqn (1). We have included an expanded ^1^H NMR spectrum in Fig. S6 (refer to SM) showing the α-CH_2_ signal (triplet) adjacent to the carbonyl group of unreacted remaining oleic acid (–CH_2_–COOH). This signal appears with very low intensity, consistent with the presence of only a trace amount of unreacted oleic acid (<5%). This observation confirms that the minor residual oleic acid detected by GC-MS. The characteristic –COOH proton (∼10.5–12 ppm) is broad, exchangeable, and often too weak to be observed at low concentrations. ^1^H NMR cannot easily differentiate the unreacted acid (<5%) from the corresponding ester (96.61%) at this position. The ^13^C-NMR spectrum given in Fig. 4b confirmed the formation of methyl oleate, as evidenced by the characteristic signals at *δ* 174.34 (carbonyl carbon, –COOCH_3_), *δ* 129.75 and 130.00 (olefinic carbons, –CH=CH–), and *δ* 51.45 (methoxy carbon, –OCH_3_).

**Fig. 5:**
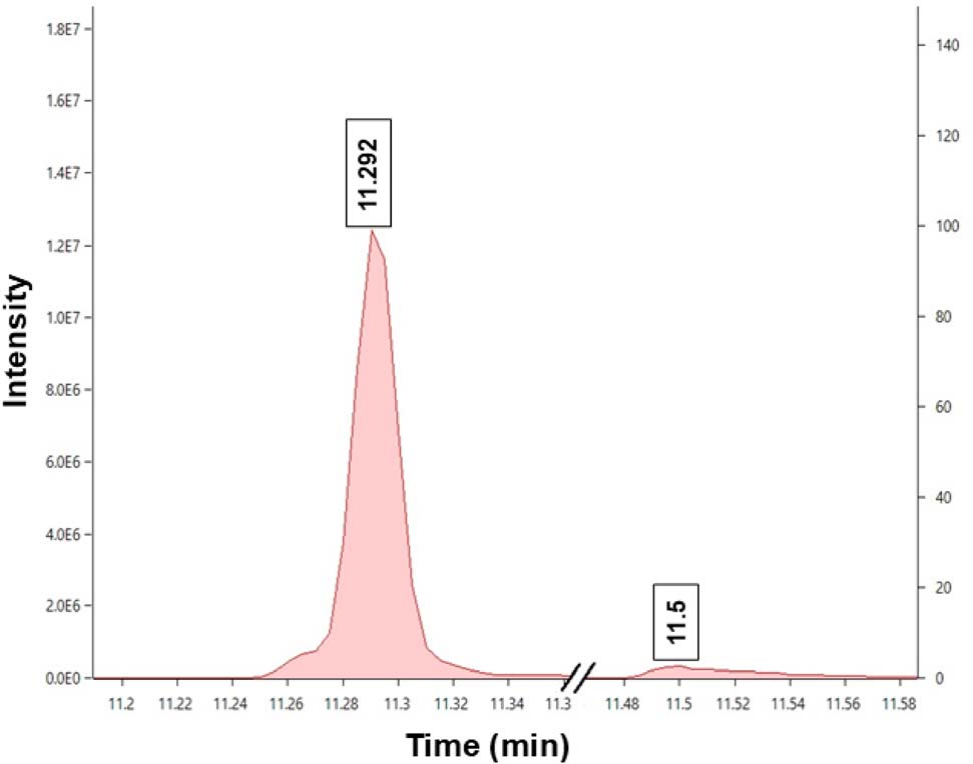
GC of biodiesel (methyl oleate) eluted under the conditions given in Fig. S4.

The chemical composition of the biodiesel was analysed using GC-MS, as presented in Fig. 5 and Table 1. According to the analysis, the major FAME identified was methyl (*E*)-9-octadecenoate (methyl oleate biodiesel), exhibiting a conversion efficiency of 92.94%, as determined from eqn (3). The excluded minor impurity (as shown in Fig. 5) marginally affects the yield; excluding it enhances the conversion efficiency to nearly 95%, which shows good agreement with the NMR-derived conversion value (96.61%). Mass spectra of methyl oleate biodiesel are given in Fig. S2 (refer to SM). The yield of biodiesel, as determined by eqn (2), was 98.61%.

**Table 1:** Compositions of biodiesel (methyl oleate)

R time	Area	Area %	Name
11.292	15575496	92.94	9-Octadecenoic acid, methyl ester, (*E*)-
11.500	1182352	7.06	Oleic acid

A plausible mechanism for the esterification reaction catalyzed by CTF-POP-SO_3_H is illustrated in Fig. S5 (refer to SM). Detailed analysis of mechanistic pathway and microwave irradiation synergy effect is given in SI.

### 3.3. Optimization of methyl oleate from oleic acid reaction

In this study, a single factor (one variable at a time) optimization approach was employed which is given in Table S1 (refer to SM). This method was chosen to individually assess the influence of key parameters such as catalyst loading, temperature, methanol-to-oil molar ratio, and reaction time on the esterification efficiency.^37^ Each experiment was performed three times then calculated the mean ± standard deviation and results are reported in Table S1 (refer to SM), along with error bars in the Fig S3 (refer to SM), to ensure statistical reliability. The equations eqn (S1) and (S2) (refer to SM) were used to calculate error bars for each optimization.

#### 3.3.1. Catalyst loading

The optimal reaction conditions of a 20 : 1 MOMR, 100 °C temperature, and 50 min time were achieved by increasing the catalyst loading from 2 to 10 wt%. As expected, oleic acid conversion increased as catalyst quantity increased, peaking at 96.61% at 8 wt%, as shown in Fig. S3a (refer to SM). A minor reduction to 93.7% was observed at 10 wt%, which may be attributed to product buildup on the catalyst surface or blockage of its active sites. Moreover, the higher catalyst concentration could have increased the reaction mixture's viscosity, introducing limitations in mass transfer.^38^

#### 3.3.2. Reaction temperature

While keeping the other parameters constant, the reaction temperature was adjusted between 40 and 120 °C, 8 wt% catalyst, MOMR of 20 : 1, and a 50 min reaction time. Every experiment was conducted in a pressure tube with a 50 W microwave irradiation at 100 psi (6.8 bar). Both transesterification and esterification are heat-absorbing processes; thus, increasing the temperature initially enhanced conversion.^39^ A maximum value of 96.61% was observed at 100 °C as shown in Fig. S3b (refer to SM), signifying this as the optimal temperature. A slight reduction in conversion at 120 °C is probably attributable to methanol loss *via* evaporation, resulting in a reduced concentration within the mixture.

#### 3.3.3. Methanol to oleic acid molar ratio

Standardized conditions of 8 wt% catalyst loading (in relation to oleic acid) and a temperature of 100 °C for 50 min were used to analyse the impact of the methanol-to-oleic acid molar ratio (MOMR) on the esterification reaction. MOMR values between 5 : 1 and 25 : 1 was examined. As the methanol concentration increased, the reaction equilibrium shifted toward ester formation, leading to improved biodiesel yield. A peak conversion of 96.61% was attained at an MOMR of 20 : 1 as shown in Fig. S3c (refer to SM). Interestingly, further increasing the ratio to 25 : 1 resulted in a slight reduction in conversion to 91.7%. This decrease can be attributed to the excessive methanol diluting the concentration of oleic acid, the limiting reactant, which reduces the likelihood of effective collisions between reactants and active centres of the catalyst.^40^ Moreover, the accumulation of water a byproduct of the esterification process can further hinder the reaction by shifting the equilibrium backward, thus slightly lowering the overall conversion rate.

#### 3.3.4. Reaction time

To evaluate the influence of reaction time on oleic acid transformation, the duration was varied between 20 and 60 min while keeping the catalyst 8 wt%, MOMR at 20 : 1 and temperature fixed at 100 °C. A peak transformation rate of 96.61% was attained in 50 min as shown in Fig. S3d (refer to SM), after which the conversion rate stabilized.^41^ Therefore, the ideal reaction parameters under microwave irradiation were determined to be an MOMR ratio of 20 : 1, 8 wt% catalyst loading, a temperature of 100 °C, and a reaction duration of 50 min. However, the ideal reaction conditions (8 wt% catalyst loading, MOMR 20 : 1, 100 °C, and 50 min reaction time) only produced 55.5% conversion from oleic acid to methyl oleate when heated conventionally in an oil bath. This proved that microwave irradiation is more effective than traditional heating techniques at speeding up the rate of reaction. Additionally, a control experiment performed under identical microwave conditions but without the catalyst afforded only a 4% conversion of oleic acid to biodiesel, confirming the essential role of the catalyst in the reaction.

### 3.4. Esterified oleic acid's kinetics and thermodynamics

**Fig. 6:**
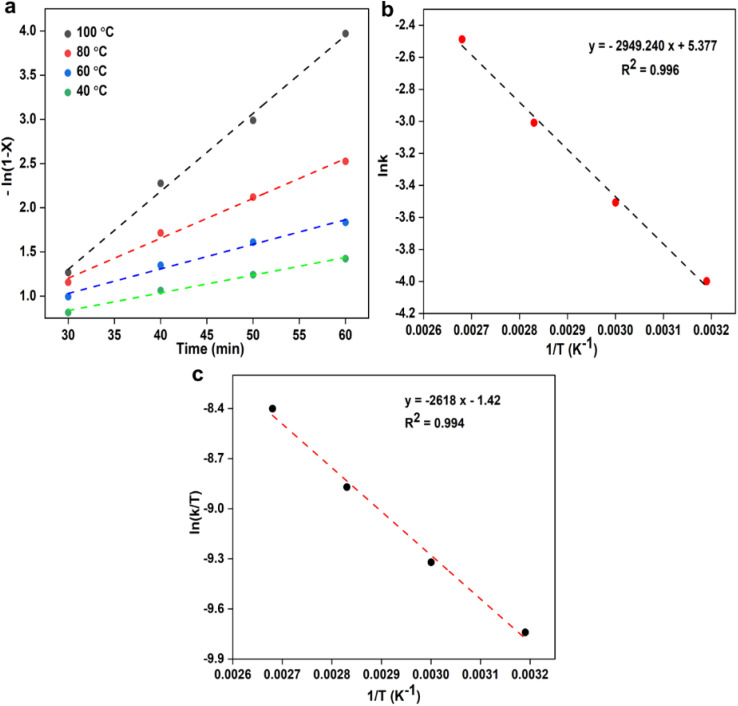
(a) −ln(1 − *X*) *vs.* time (where *X* is the yield of methyl oleate), (b) ln *k vs.* 1/*T* Arrhenius plot and (c) ln(*k*/*T*) *vs.* 1/*T* (Eyring–Polanyi plot).

For the esterification reactions performed within the temperature range of 40 to 100 °C, small aliquots of the reaction mixture were withdrawn at regular time intervals and analyzed to determine the fractional conversion (*X*) of oleic acid as depicted in Fig. 6a, time and −ln(1 − *X*) show a straight line, indicating that the esterification of OA can be explained by a pseudo-first-order kinetic model.^42^ For the esterification reaction, *E*_a_ (activation energy) was calculated by applying the Arrhenius equation, as shown in eqn (7). By plotting a graph in Fig. 6b between ln *k vs. T*^−1^, activation energy was found to be 24.52 kJ mol^−1^, and the frequency factor was found to be 2.18 × 10^2^ min^−1^. The slope and intercept of Fig. 6c, corresponding to eqn (8), were used to determine the activation parameters. The enthalpy and entropy of activation (Δ*H*^‡^ and Δ*S*^‡^) were calculated to be 21.78 kJ mol^−1^ and −209.35 J K^−1^ mol^−1^, respectively. To calculate Δ*G*^‡^ throughout the temperature range of 313–373 K, the computed values of Δ*H*^‡^ and Δ*S*^‡^ were entered into eqn (9). The negative Δ*S*^‡^ and positive Δ*H*^‡^ values indicate that esterification of OA is heat-absorbing and proceeds with a decrease in disorder.^2^ Furthermore, the reaction was found to be non-spontaneous at all temperatures, as evidenced by the positive Δ*G*^‡^ values given in Table 2.

**Table 2:** Thermodynamic studies of esterification of oleic acid using CTF-POP-SO_3_H

Temperature (K)	Δ*G*^‡^ (kJ mol^−1^)	Δ*H*^‡^ (kJ mol^−1^)	Δ*S*^‡^ (J K^−1^ mol^−1^)
313 K	87.11	21.78	−209.35
333 K	91.39		
353 K	95.68		
373 K	99.87		

### 3.5. Comparison with reported heterogeneous catalysts

Recent studies have reported a diverse range of heterogeneous catalysts, including porous organic polymers (POPs), covalent organic frameworks (COFs), metal–organic frameworks (MOFs), and supported metal oxides for the environmentally friendly production of biodiesel. Table 3 presents a comparative summary of key parameters such as catalyst type, feedstock, operating conditions, and biodiesel yield for benchmarking our developed catalyst against reported systems. Among the reported catalysts, PPM-SO_3_H,^43^*x*AIL@TpPa-SO_3_H,^44^ MnO_2_@Mn(btc),^45^ and WP-SO_3_H-6 ^46^ required longer reaction times to afford biodiesel compared to present work. Our present work gave high biodiesel yield and conversion percentage at very minimum reaction time compared to other catalysts reported in the Table 3.

**Table 3:** Comparison of various solid heterogeneous catalysts used in biodiesel synthesis

Sl. no	Catalyst	Feedstock	Reaction conditions^a^	Yield (%)	Ref.
1	PPM-SO_3_H(POP)	Waste fatty acid	1 : 1, 20%, 80 °C, 8 h	96.9	43
2	*x*AIL@TpPa-SO_3_H (COF)	Soybean oil	30 : 1, 10%, 120 °C, 8 h	93.9	44
3	UiO-66/SA (MOF)	Oleic acid	21.9 : 1, 7.6%, 85 °C, 1.8 h	96.4	2
4	MnO_2_@Mn(btc)	Oleic acid	12 : 1, 3%, 100 °C, 12 h	98	45
5	WP-SO_3_H-6	Oleic acid	20 : 1, 8%, 100 °C, 20 h	94.44	46
6	Mo-MOF	Oleic acid	13 : 1, 30%, 60 °C, 4 h	95	47
7	ZIF-8/TiO_2_	Oleic acid	30 : 1, 6%, 50 °C, 1.04 h	80.04	48
8	AIL@NH_2_-UiO-66	Oleic acid	14 : 1, 5%, 75 °C, 2 h	97.52	49
9	CTF-POP-SO_3_H	Oleic acid	20 : 1, 8%, 100 °C, 50 min	98.61^b^	(This work)
				96.61^c^	

a MOMR, catalyst loading, reaction temperature, reaction time.

b Yield.

c Conversion.

### 3.6. Heterogeneous test and catalysts' reusability

**Fig. 7:**
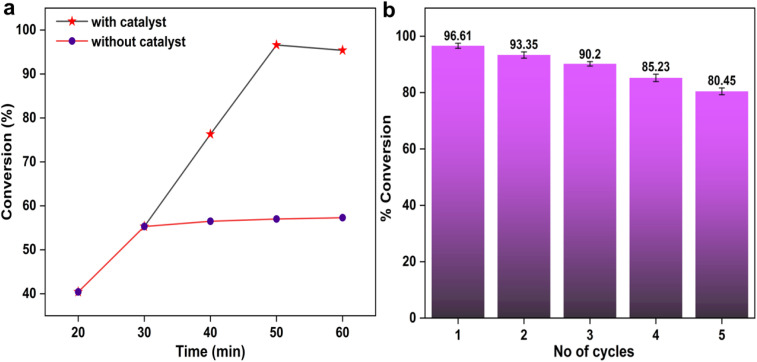
(a) Biodiesel conversion obtained using CTF-POP-SO_3_H and the reaction conditions MOMR 20 : 1, 100 °C temperature, 8 wt% catalyst, and time 20–60 min. (black line) and after removal of catalyst at 30 min. and continuation of reaction without catalyst (red line). (b) Reusability of the CTF-POP-SO_3_H catalyst over five esterification cycles.

A heterogeneity test on the produced CTF-POP-SO_3_H catalyst was carried out using the hot filtering method (Sheldon's test).^50^ As shown in Fig. 7a, the catalyst was separated by filtering at a high temperature after a 30 min reaction period, yielding a 55.3% yield. The process was then extended for another 30 min without the catalyst, producing a product yield of 57.3%. This investigation verified that the filtrate's soluble active species concentration was so low that it was unable to enhance catalytic activity any further. Thus, it amply demonstrated the catalyst's heterogeneous nature.

After every cycle, the CTF-POP-SO_3_H catalyst was extracted from the reaction mixture by filtering it out to determine its recyclability. After being recovered, the catalyst was cleaned with methanol by centrifugation and facilitated to dry for 4 h at 90 °C in an oven. Weighing the catalyst before reuse allowed us to track any mass loss. Following that, the catalytic reaction was carried out four more times in a row using the previously adjusted conditions, using the same recovery procedure each time. After the fifth catalytic cycle, SEM-EDX analysis was taken as shown in Fig. S5 (refer to SM), indicating a reduction in sulfur content in the recovered CTF-POP-SO_3_H catalyst, suggesting partial leaching of active sites during the washing and activation steps.^51^ Consequently, a gradual decline in catalytic performance was observed, Fig. 7b shows that oleic acid's transformation into methyl oleate biodiesel decreased from 96.61 ± 0.7% in the first cycle to 80.45 ± 0.9% by the fifth cycle.

### 3.7. Bibliometric insights

**Fig. 8:**
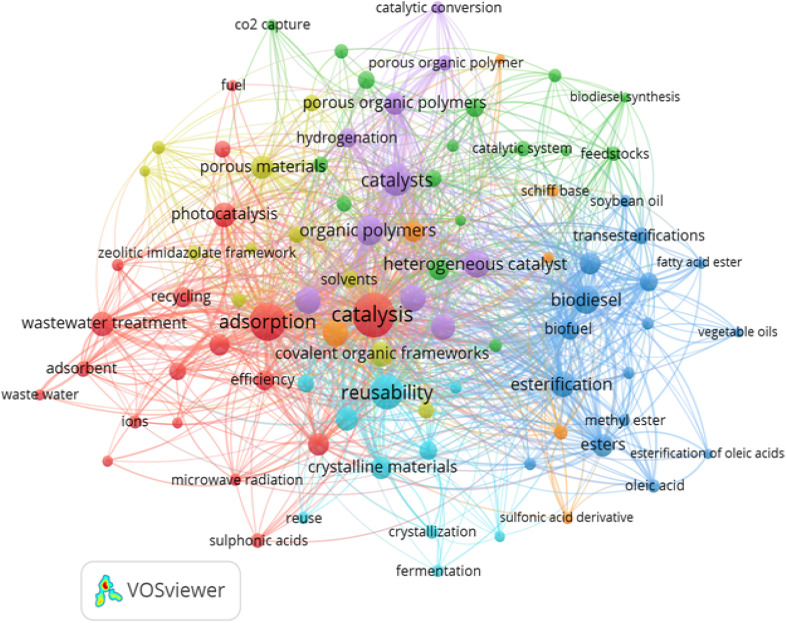
Bibliometric study of porous organic polymers.

As a research approach, bibliometrics offers a thorough summary and organized categorization of previous and ongoing studies, facilitating deeper understanding and assisting in the identification of future research trends.^52^ The knowledge map presented in Fig. 8 was generated using VOSviewer analysis, based on up to 200 publications sourced from the Scopus Collection databases. The analysis focused on key terms such as porous organic polymer; biodiesel synthesis and catalysis applying the fractional counting method to illustrate and trace developments in the field. This mapping approach helps researchers gain meaningful insights into ongoing trends, upcoming challenges, and dominant areas of interest in their domain.^53,54^ The yellow cluster in Fig. 8 focuses on the design and application of advanced porous materials, especially COFs, porous organic polymers, and hybrids. Because of their adjustable pore structure and functionalizable frameworks, porous organic polymers (POPs) are perfect surfaces for designing heterogeneous catalysts. The blue cluster focuses on the esterification process of oleic acid and biodiesel production from vegetable oils. It highlights converting FFAs like oleic acid into FAMEs to overcome challenges with high FFA feedstocks.

In the green cluster, the central theme is creating a sustainable and integrated platform for biodiesel synthesis and CO_2_ mitigation. Thus, POPs and their application (biodiesel production and CO_2_ capture) are intricately linked, reflecting a unified research strategy toward green and sustainable chemical processes. The red cluster focuses on the multifunctional use of porous materials, such as sulfonic acid-functionalized POPs and COFs for catalysis, adsorption, and energy-related applications. Overall, the cluster highlights the synergy between material design (porosity, –SO_3_H groups) and performance for sustainable fuel synthesis and environmental protection.

## 4. Life cycle cost analysis

Life Cycle Cost Analysis (LCCA) is a thorough approach to assessing the total cost of a process or product over the course of its whole life cycle.^3^ Important factors that affect the overall cost of production in biodiesel synthesis include the kind of catalyst, energy usage, waste management, and feedstock selection. LCCA makes it possible to conduct a thorough evaluation of the sustainability and economic viability of biofuel production by contrasting the lifetime costs of biodiesel with those of traditional fossil fuels.

The LCCA was conducted in two phases in this study. The first step was to figure out how much it would cost to make the porous organic polymer catalyst CTF-POP-SO_3_H given in Table S2 (refer to SM). To make 1 kg of biodiesel, we need 77.1 g of catalyst. The total cost of making 77.1 g of catalyst was $3.66 USD. The catalyst can be used again for five reaction cycles, which brought the effective cost of the catalyst for making biodiesel down to $0.732 USD per kg of biodiesel. Second, it was calculated that it would cost $2.182 USD per kg (Table S3, refer to SM) to make 1 kg of biodiesel (methyl oleate) from oleic acid feedstock using the CTF-POP-SO_3_H catalyst under microwave-assisted esterification conditions (MOMR 20 : 1, 100 °C temperature, 8 wt% catalyst, 50 min reaction time). This comprises the expenses of electricity, feedstock, methanol, and the catalyst. The increased value is mostly because pure oleic acid is used, which costs a lot more than low-grade feedstocks.

This laboratory-scale calculation may not accurately represent industrial settings, but it offers a plausible cost approximation. The findings show that CTF-POP-SO_3_H is both economically viable and reusable. This makes it a cleaner and more sustainable alternative to traditional homogeneous catalysts, which can't be recovered and create a lot of effluent. In general, the technology shows a lot of promise for making biodiesel in the future from waste lipid feedstocks.

Future research will investigate the substitution of refined oleic acid with waste cooking oil (WCO) or *Jatropha curcas* oil (JCO). These feedstocks that cost nothing or are waste materials are projected to lower the total manufacturing cost a lot since they don't cost much or anything at all for raw materials. This kind of optimization could lower the overall cost of biodiesel production down to less than $1 USD per kg.

## 5. Scale-up challenges and economic prospects of biodiesel production

There are both technological and financial obstacles to overcome when increasing biodiesel production from experimental to industrial levels. Large-scale implementation necessitates significant capital investment in advanced reactor design, process automation, energy-management systems, and environmental compliance infrastructure, all of which raise overall operating costs.^55^ It is important to recognize the challenges associated with scaling up biodiesel production, even though the present LCCA provides valuable insights at the laboratory scale. Among all variables, feedstock cost remains the dominant factor, contributing up to 80% of the total biodiesel production cost.^56^ Implementing a continuous transesterification process and extracting high-purity glycerol as a valuable by-product are efficient ways to drastically lower the total costs of producing biodiesel when waste cooking oil is utilized as the feedstock.

Biodiesel has a lot of economic benefits, such as being more energy-efficient, being able to break down naturally, perhaps helping to slow down global warming, reducing the need for crude oil imports, and creating jobs in the agriculture sector. Biodiesel as a sustainable fuel is even more appealing now that people are more concerned of the environment. Most diesel engines and the current infrastructure for storage and distribution can run on mixes of biodiesel and petroleum fuel up to 20% (B20).^57^ Furthermore, geographical differences in the cost of power and methanol may have a big impact on economic viability. Future research should include sensitivity analyses to assess the effect of feedstock and utility price variations on overall production economics in order to strengthen the robustness and practical usefulness of LCCA. These evaluations are necessary to guarantee biodiesel's long-term sustainability and competitiveness as a substitute energy source.

## 6. Conclusion

In this work, we successfully synthesized the porous organic polymer CTF-POP by introducing sulfonic acid groups using chlorosulfonic acid to give CTF-POP-SO_3_H for biodiesel synthesis, which yielded 98.6 ± 0.6% methyl oleate with a conversion rate of 96.61%. Comprehensive physicochemical characterization of the catalyst was carried out using XRD, SEM-EDX, BET, XPS, TGA, and FT-IR. Sulphonic acid group density of CTF-POP-SO_3_H was found to have 0.21 mmol g^−1^ by acid–base titration. This sulfonation boosted the material's acidity, making the resulting CTF-POP-SO_3_H a more effective catalyst for converting oleic acid into biodiesel. The use of an acidic catalyst combined with microwave-assisted heating significantly accelerated the transformation of oleic acid to methyl oleate, achieving high reaction rates with activation energy of 24.52 kJ mol^−1^ at 100 °C reaction temperature and 8 wt% catalyst loading in a much shorter time (50 min) compared to conventional methods. Furthermore, CTF-POP-SO_3_H exhibited good reusability, maintaining above 80% yield over five cycles without significant loss of activity. These results demonstrate the CTF-POP-SO_3_H catalyst's potential as a viable and sustainable catalyst for the synthesis of biodiesel from free fatty acids.

The LCCA demonstrated its economic viability by demonstrating that, from a techno-economic perspective, the catalyst's effective cost per kg of biodiesel was just $0.732 USD. Owing to its metal-free composition and excellent reusability, the system is both environmentally benign and economically sustainable. Future work is directed toward scaling up the synthesis process, enhancing acid-site stability, and extending the catalyst's applicability to real waste oil feedstocks to enable sustainable large-scale biodiesel production.

## Author contributions

Biman Kaushik: writing – original drafts, software, methodology, formal analysis, data curation, conceptualization. Shikhasmita Das: writing – review & editing. Sanjay Basumatary: writing – review & editing. Ruma Rano: writing – review & editing. Hui Li: writing – review and editing. Jasha Momo H. Anal: writing – review & editing. Gopinath Halder: writing – review & editing. Samuel Lalthazuala Rokhum: conceptualization, writing – review & editing, investigation, data, formal analysis, and supervision.

## Conflicts of interest

There are no conflicts to declare.

## Abbreviations

BET: Brunauer–Emmett–Telle
MOMR: Methanol to oil molar ratio
BJH: Barrett–Joyner–Halenda analysis
NMR: Nuclear magnetic resonance
CTF-POP: Covalent triazine framework porous organic polymer
OA: Oleic acid
CTF-POP-SO_3_H: Sulfonated covalent triazine framework porous organic polymer
POP: Porous organic polymer
DCM: Dichloromethane
R-CTF-POP-SO_3_H: Recovered sulfonated covalent triazine framework porous organic polymer
EDX: Energy dispersive X-ray spectroscopy
SM: Supplementary material
FAME: Fatty acid methyl esters
SEM: Scanning electron microscopy
FFA: Free fatty acid
TGA: Thermo gravimetric analysis
FT-IR: Fourier Transform Infrared Spectroscopy
XPS: X-ray photoelectron spectroscopy
GC-MS: Gas chromatography-mass spectrometry
XRD: X-ray diffraction
MO: Methyl oleate
XPS: X-ray photoelectron spectroscopy

## Supplementary Material

RA-015-D5RA07846F-s001

## Data Availability

The data will be made available from the corresponding author upon reasonable request. Supplementary information (SI) is available. See DOI: https://doi.org/10.1039/d5ra07846f.
