# Supplementary material for: One-step, efficient and sustainable microwave-assisted biodiesel production using a sulfonated porous organic polymer catalyst
Source: RSC Adv. 2025 Nov 27;15(55):46843–55. doi: 10.1039/d5ra07846f (PMC12659726; doi:10.1039/d5ra07846f)
Supplement: RA-015-D5RA07846F-s001 [file RA-015-D5RA07846F-s001.pdf]

Supplementary Information (SI) for RSC Advances.
This journal is © The Royal Society of Chemistry 2025

Supplementary Information

One-Step, Efficient and Sustainable Microwave-Assisted Biodiesel Production Using a Sulfonated Porous Organic Polymer Catalyst

*Biman Kaushik [a], Shikhasmita Das [a], Sanjay Basumatary [b], Ruma Rano [a], Hui Li [c], Jasha Momo H. Anal [d], Gopinath Halder [e], Samuel Lalthazuala Rokhum [a]**

[a] *Department of Chemistry, National Institute of Technology Silchar, Silchar 788010, Assam, India*

[b] *Department of Chemistry, Bodoland University, Kokrajhar, 783370, Assam, India*

[c] *School of Thermal Engineering, Shandong Jianzhu University, Jinan 250101, PR China*

[d] *Natural Products and Medicinal Chemistry Division, CSIR-Indian Institute of Integrative Medicine, Jammu, Jammu and Kashmir, 180001 India*

[e] *Department of Chemical Engineering, National Institute of Technology Durgapur, Durgapur 713209, West Bengal, India*

Corresponding author's email: rokhum@che.nits.ac.in (SLR)

**Sl. No**	**Caption**	**Figures**
1	BET and pore size distribution of recovered catalyst	**Fig. S1**
2	Mass spectra of bio-diesel (methyl oleate)	**Fig. S2**
3	Reaction parameters influence on biodiesel conversion	**Fig. S3**
4	SEM-EDS spectrum of reused catalyst	**Fig. S4**
5	Single-factor optimization of biodiesel using CTF-POP-SO_3H catalyst.	**Table S1**
6	General plausible mechanism of esterification of oleic acid	**Fig. S5**
7	An expansion of the α-CH_2 region showing triplets due to minor residual starting material (OA) at 2.37 ppm	**Fig. S6**
8	Cost of catalyst for 1 kg biodiesel	**Table S2**
9	Cost of 1 kg biodiesel from oleic acid by using microwave irradiation	**Table S3**

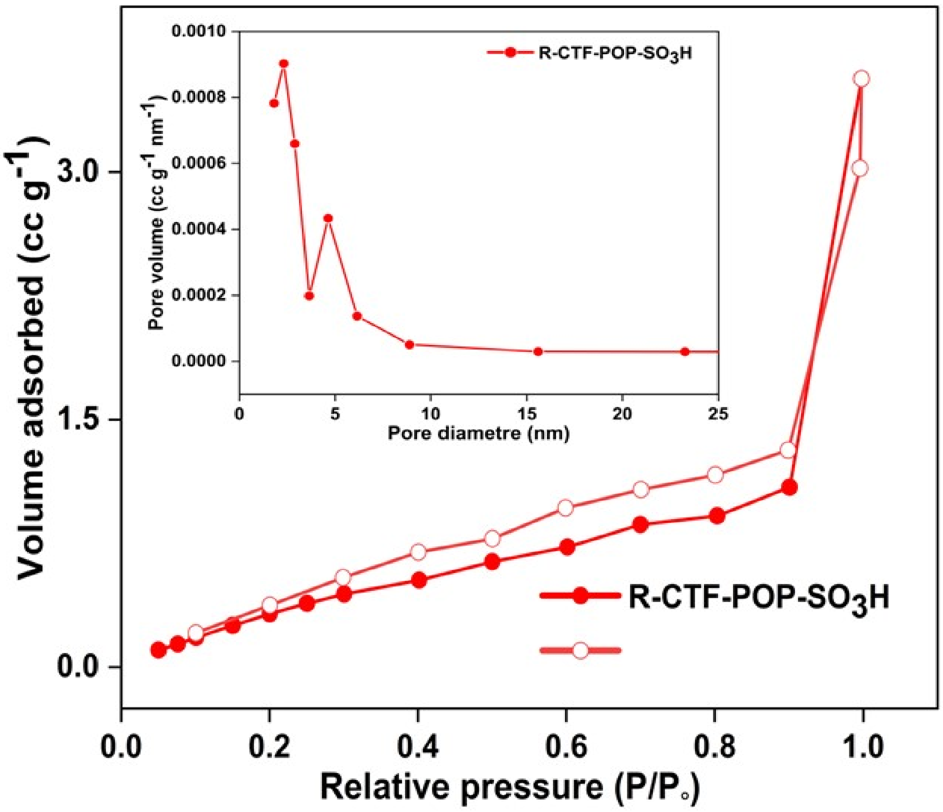

Fig. S1: BET and pore size distribution of recovered catalyst (R-CTF-POP-SO_3H)

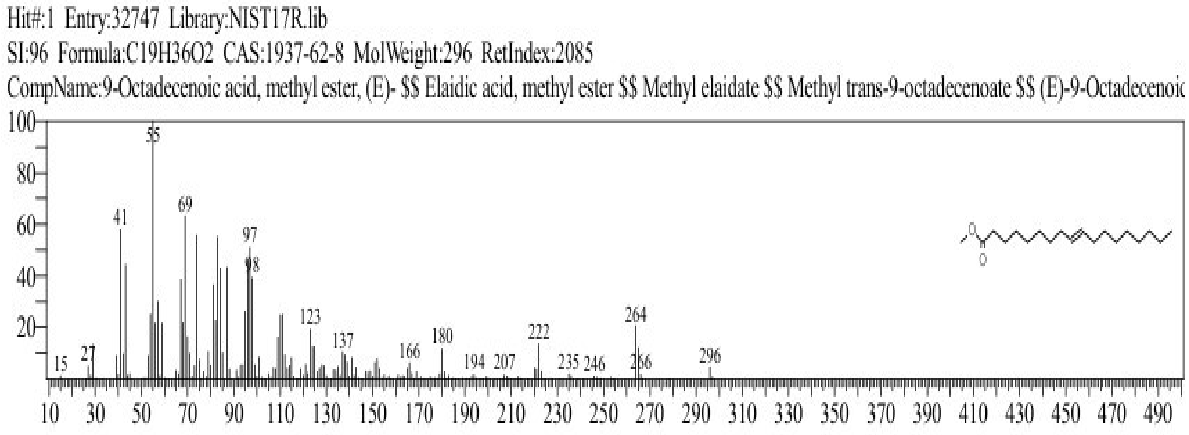

Fig. S2: Mass spectra of biodiesel (methyl oleate) eluted under optimized conditions.

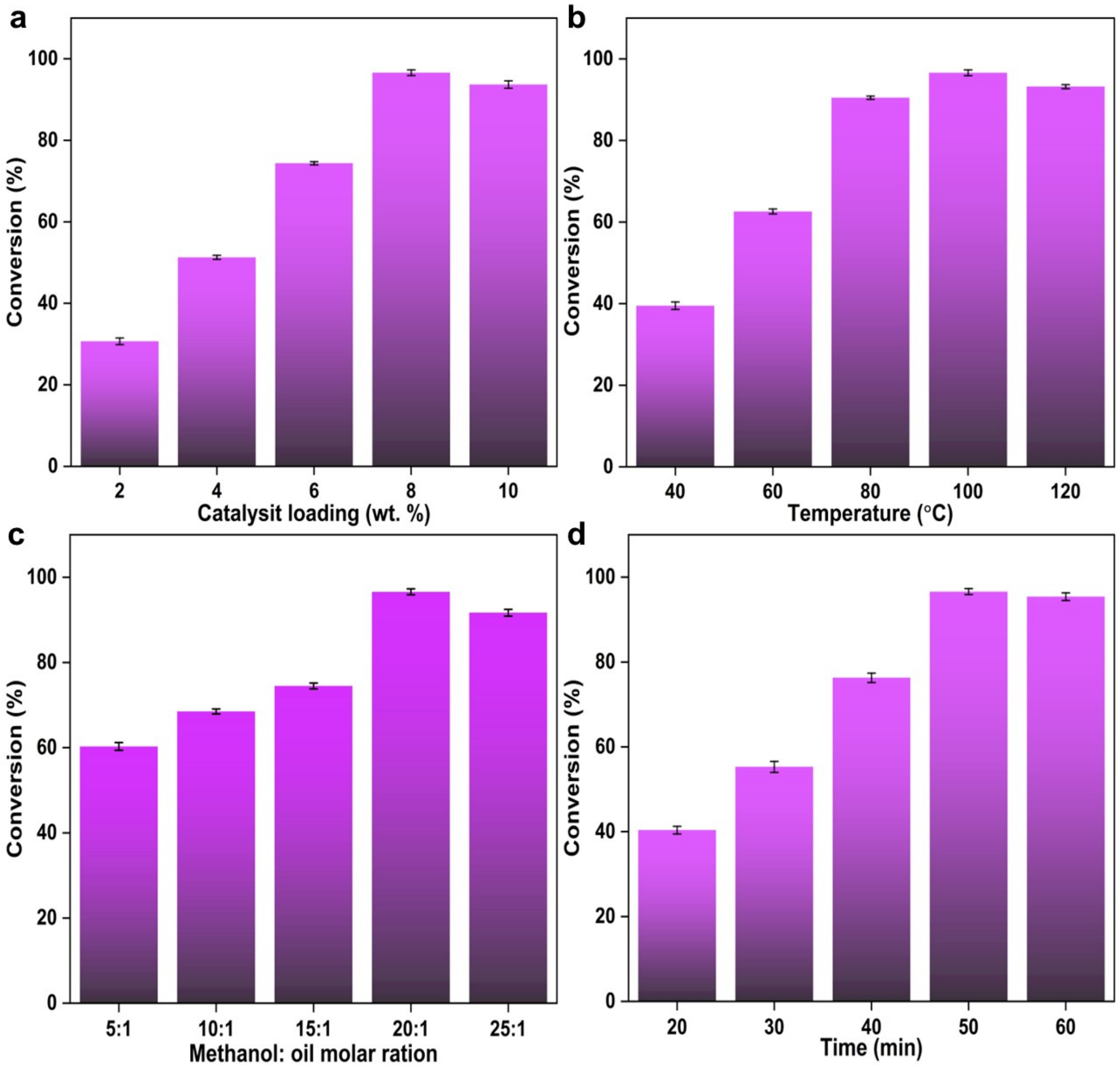

Fig S3: Reaction parameters influence on biodiesel conversion-(a) Catalyst loading, (b) Temperature, (c)MOMR, (d) reaction time.

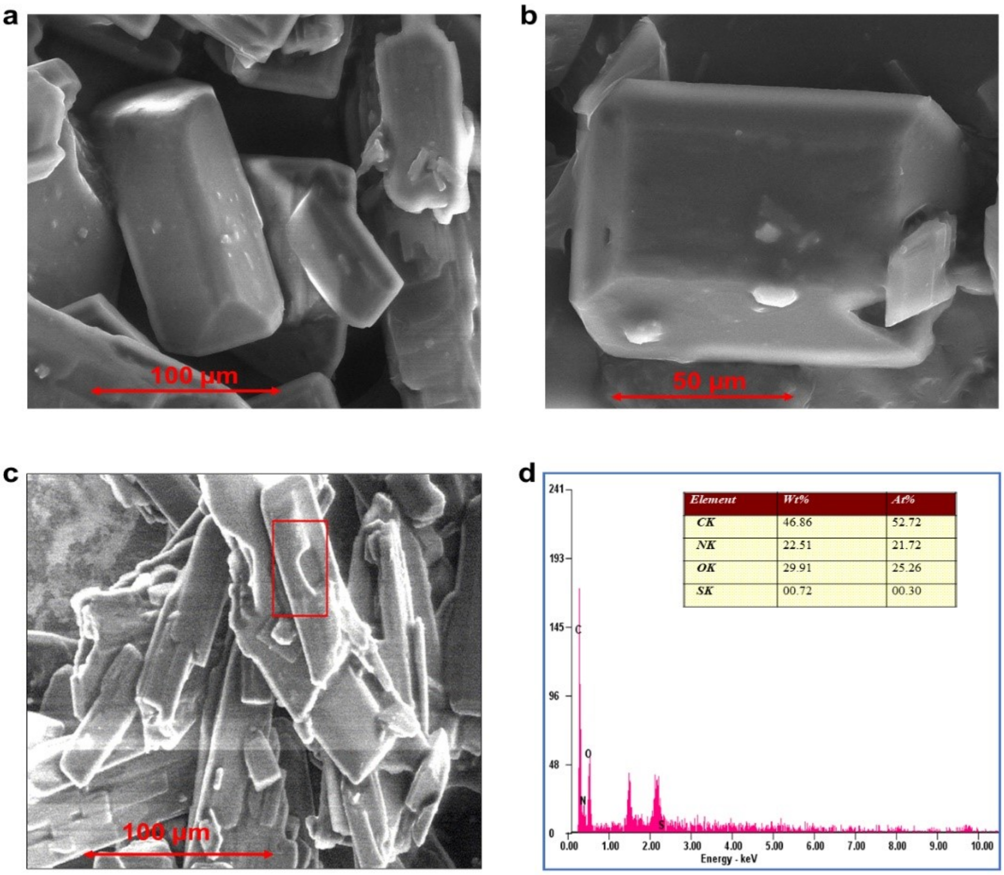

Fig. S4: SEM images of (a-c) recovered catalyst R-CTF-POP-SO_3H along with the EDX data (d) for the region highlighted in the red box in (c).

Table S1. Single-factor optimization of biodiesel using CTF-POP-SO_3H catalyst.

Catalyst loading (wt. %)	Temperature (°C)	Methanol to Oil Ratio	Time (min)	Conversion (%)
2	100	20:1	50	30.7 ± 0.8
4	100	20:1	50	51.3 ± 0.5
6	100	20:1	50	74.4 ± 0.4
8	100	20:1	50	96.6 ± 0.7
10	100	20:1	50	93.7 ± 0.9
8	40	20:1	50	39.5 ± 0.9
8	60	20:1	50	62.6 ± 0.6
8	80	20:1	50	90.5 ± 0.4
8	100	20:1	50	96.6 ± 0.7
8	120	20:1	50	93.2 ± 0.5
8	100	5:1	50	60.3 ± 0.9
8	100	10:1	50	68.5 ± 0.6
8	100	15:1	50	74.5 ± 0.7

8	100	20:1	50	96.6 ± 0.7
8	100	25:1	50	91.7 ± 0.8
8	100	20:1	20	40.4 ± 0.9
8	100	20:1	30	55.3 ± 1.3
8	100	20:1	40	76.3 ± 1.1
8	100	20:1	50	96.6 ± 0.7
8	100	20:1	60	95.4 ± 0.9

$$Mean(\grave{x}) = \frac{\sum x_i}{n}$$ **Eq. S1**

Eq. S2

$$SD = \sqrt{\frac{\sum (x_i - \bar{x})^2}{n-1}}$$

where n = no of experiments. x_i= each value. $\grave{x}$ = mean for one optimized parametre.

SD = Standard Deviation

At the optimum condition (8 wt % catalyst loading, 100 °C temperature, 20: 1 MOMR, 50 min reaction time), the reaction was performed three times. So, for this we got 96.6 % mean and 0.7 % standard deviation.

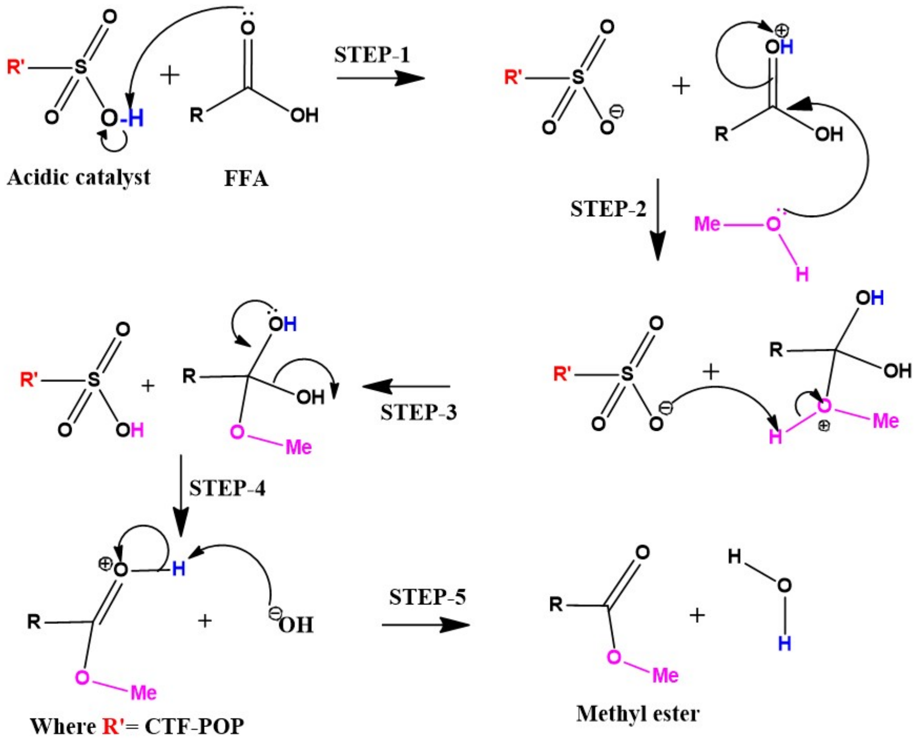

Fig. S5: Esterification of free fatty acid (oleic acid) to methyl ester (methyl oleate).

Mechanistic pathway:

The plausible reaction mechanism for the microwave-assisted esterification of oleic acid with methanol using CTF-POP-SO_3H as catalyst is illustrated in **Fig. S5** and proceeds through the following elementary steps:

1. Activation of Oleic Acid:
   The Brønsted acidic -SO_3H sites of the CTF-POP-SO_3H catalyst protonate the carbonyl oxygen of oleic acid (R-COOH), thereby increasing the electrophilicity of the carbonyl carbon.
2. Activation of Methanol and nucleophilic attack:
   Methanol is activated through hydrogen bonding and subsequently attacks the protonated carbonyl carbon to form a tetrahedral intermediate.
3. Catalyst Regeneration:
   The -SO_3H site is regenerated by releasing the proton consumed in the first step.
4. Proton Transfer and Water Elimination:
   Step 4 and 5 involves proton transfer and the elimination of water, leading to the formation of methyl ester, thus completing the catalytic cycle.

Microwave-Assisted synergy: Microwave irradiation speeds up the process by heating the whole volume evenly via the dipolar rotation of methanol and the polarization of sulfonic acid groups. This microwave-acidic-site synergy speeds up proton transfer and the creation of intermediates, leading to full conversion in 50 minutes, which is much quicker than traditional thermal approaches.

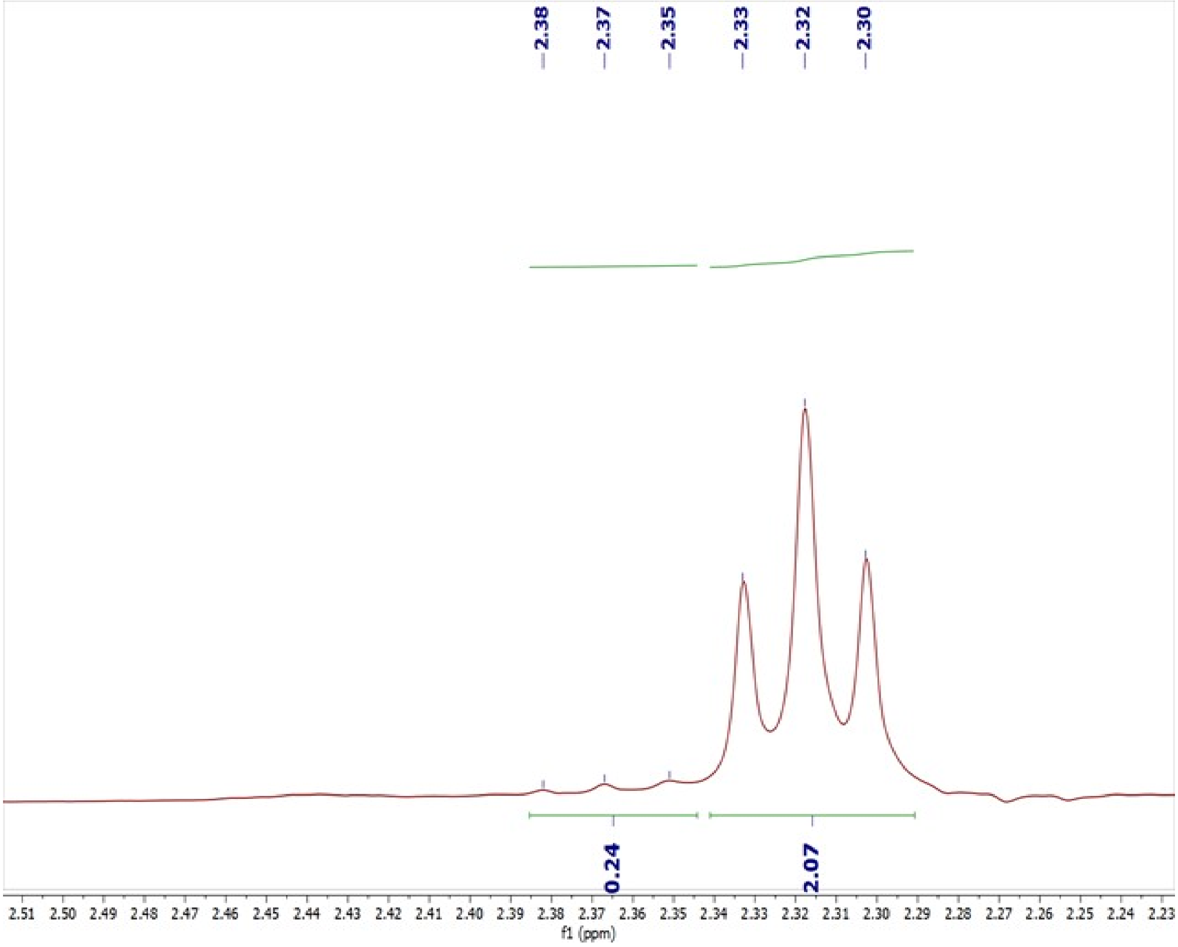

Fig S6: An expansion of the α-CH_2 region showing triplets due to minor residual starting material (OA) at 2.37 ppm

Table S2: Cost of catalyst for 1 kg biodiesel

SL. No.	Name of chemical	Req. amount for 2.5 g catalyst	Req. amount for 77.1 g catalyst	[a]Unit price (USD)	Total price (USD)
1	Anhydrous $AlCl_3$	2.6 g	80.184 g	0.45/kg	0.036

2	Cyanuric chloride	1 g	30.84 g	1.41/kg	0.043
3	Triphenyl methane	1.3 g	40.092 g	1.13/kg	0.045
4	DCM	61 mL	1.881 L	0.37/L	0.696
5	THF	4 mL	123.36 mL	1.52/L	0.188
6	Methanol	16 mL	493.44 mL	0.12/L	0.059
7	Chlorosulphonic acid	1.5 mL	46.26 mL	1.09/L	0.05
8	Electricity	1.5 unit	46.26 unit	0.055/unit	2.544
9	Total amount				3.661
10	Reusability 5 cycles				3.661/5
11	Total Cost of catalyst				**0.732**

[a]Cost of raw materials are as per the purchased quotation price from Sisco research lab, BLD pharma, India Mart, Trade India, and Scientific global Guwahati, India. 1USD = 88.7 (Nov. 2025)

61

Table S3: Cost of 1 kg biodiesel from oleic acid by using microwave irradiation

SL. NO	Process	Description	Unit price (USD)	Cost (USD)
1	Total amount of biodiesel production	1 kg		
2	Cost of oleic acid feedstock	967 g	0.96/1kg	0.92
3	Methanol required for biodiesel	2.78 L	0.12/1L	0.33
4	Electricity	0.04 unit	0.055/unit	0.002
5	Quantity of catalyst needed for 1 kg biodiesel	77.1 kg		0.732

6	Total cost		1.984
	Overhead Cost	10 % net charge	0.198
	Total cost for 1 kg biodiesel		**2.182**
